# The Fungal Microbiome of Wheat Flour Includes Potential Mycotoxin Producers

**DOI:** 10.3390/foods11050676

**Published:** 2022-02-25

**Authors:** Serena A. Minutillo, David Ruano-Rosa, Ahmed Abdelfattah, Leonardo Schena, Antonino Malacrinò

**Affiliations:** 1 CIHEAM—Centre International de Hautes Etudes Agronomiques Méditerranéennes, Mediterranean Agronomic Institute of Bari, 70010 Valenzano, Italy; minutillo@iamb.it; 2 Instituto Tecnológico Agrario de Castilla y León, Consejería de Agricultura y Ganadería, 47007 Valladolid, Spain; ruarosda@itacyl.es; 3 Leibniz-Institute for Agricultural Engineering Potsdam (ATB), University of Potsdam, 14469 Potsdam, Germany; ahmed.abdelfattah@tugraz.at; 4 Dipartimento di AGRARIA, Università Mediterranea di Reggio Calabria, 89122 Reggio Calabria, Italy; lschena@unirc.it

**Keywords:** *Penicillium*, *Alternaria*, post-harvest, metabarcoding

## Abstract

Consumers are increasingly demanding higher quality and safety standards for the products they consume, and one of this is wheat flour, the basis of a wide variety of processed products. This major component in the diet of many communities can be contaminated by microorganisms before the grain harvest, or during the grain storage right before processing. These microorganisms include several fungal species, many of which produce mycotoxins, secondary metabolites that can cause severe acute and chronic disorders. Yet, we still know little about the overall composition of fungal communities associated with wheat flour. In this study, we contribute to fill this gap by characterizing the fungal microbiome of different types of wheat flour using culture-dependent and -independent techniques. Qualitatively, these approaches suggested similar results, highlighting the presence of several fungal taxa able to produce mycotoxins. In-vitro isolation of fungal species suggest a higher frequency of *Penicillium*, while metabarcoding suggest a higher abundance of *Alternaria*. This discrepancy might reside on the targeted portion of the community (alive vs. overall) or in the specific features of each technique. Thus, this study shows that commercial wheat flour hosts a wide fungal diversity with several taxa potentially representing concerns for consumers, aspects that need more attention throughout the food production chain.

## 1. Introduction

Wheat flour is an essential ingredient for the human diet on a global scale. However, the final quality and safety of flour-based products can be greatly influenced by fungal contaminations that can occur before and/or after harvest [1,2,3]. For example, fungi belonging to the genera *Alternaria*, *Cladosporium*, *Fusarium*, and *Helmintosporium* have been reported as contaminants of grains in the field (moisture content of 18–30%), while *Aspergillus*, *Penicillium*, *Eurotium*, and *Mucor* are mainly reported to contaminate grains in storage conditions (moisture content of 14–16%) [2,4]. These fungal genera include many species that can produce mycotoxins, fungal secondary metabolites that when ingested, inhaled, or absorbed through skin, can cause both acute [5,6] and, most importantly, chronic disorders (e.g., reduced growth and development, immunosuppression, cancer) [7]. Although a large number of studies have focused on the analysis and quantification of mycotoxins in flour products [8,9,10], few information are currently available about the community of mycotoxin-producing fungi.

The presence of mycotoxin-producing fungi in grains influences all the downstream production chain as the milling process does not destroy fungi, and wheat flour may carry a significant mycological and mycotoxigenic load that can contaminate the food products at the end of the production chain [11,12]. Furthermore, several mycotoxins (e.g., ochratoxin A, fumonisin B1 and B2, zearalenone) have proven to be highly stable during thermal processing (baking, frying, cooking, steaming), procedures commonly used to obtain the final products [13,14,15,16,17]. About 700 million tons of grains are annually lost due to mycotoxin contaminations [18], causing an annual cost of several millions dollars. In this context, the prevention of fungal contamination is essential to reduce the concentration of mycotoxins in flour-based foods. However, the current available data are generally based on the isolation of the colony forming units (CFU) of total “molds” without a precise identification of fungal species and relative abundance. In some cases an approximate identification, generally up to the level of genera, has been performed according to morphological features [19].

In this study, we explored the fungal diversity associated with different types of wheat flour (types “0”, “00” and wholemeal) using both a culture-dependent (in vitro isolation of fungi) and a culture-independent method (metabarcoding targeting the fungal ITS2 region), showing that these food products host a wide diversity of fungi, several of which potentially producing mycotoxins.

## 2. Methods

### 2.1. Sampling and Study Overview

We analyzed the diversity of the fungal microbiome associated with different types of wheat flours using two different methods. Wheat flour samples were collected from commercial mills located in Calabria (southern Italy) during 2018. Samples included flour of type “00”, “0”, and wholemeal (3 samples each). All samples were collected as three subsamples of ∼1 kg from uniform bulks of flour. Flour samples were kept in sterile plastic bags at 5 °C and analysed within 5 days after sampling. Samples were then used to isolate fungi in vitro (culture-dependent method) and processed to prepare ITS-amplicon libraries for metabarcoding analyses (culture-independent method).

### 2.2. In-Vitro Isolation of Fungi and Molecular Identification

Alive fungal contaminants were isolated from our flour samples by plating them on potato dextrose agar (PDA) plates (Difco Laboratories, Detroit, MI, USA). From each individual sample, we collected three sub-samples of ∼10 g, and suspended them in 20 mL of sterile water:agar solution (0.1%). Each suspension was serially diluted up to a 1:1000 ratio in sterile water:agar solution (0.1%). Then, we plated 0.1 mL from each suspension on PDA plates amended with ampicillin and streptomycin (0.25 mg/L each, Sigma-Aldrich, St. Louis, MO, USA) to prevent bacterial growth. Plates were incubated at 20 ± 2 °C for 5 days, and then inspected to count the number of CFU/g of sample. Each colony was then isolated on PDA plate, and isolates were grouped according to the morphology of colonies on PDA and according to microscopic features (mycelium and, when available, asexual reproductive structures), yielding 34 representative isolates.

These representative isolates were identified using molecular barcoding. Each isolate was grown on Potato Dextrose Broth (PDB) (Difco Laboratories, Detroit, MI, USA) at 22 °C for 5 to 7 days according to the growth speed of each different isolate. The mycelium was collected by centrifugation, washed twice with sterile distilled water and lyophilized. DNA was then extracted from 20 mg of lyophilized tissue using the DNeasy Plant Mini Kit (Qiagen, Venlo, The Netherlands) according to the manufacturer’s instructions, and concentration and quality were measured using Nanodrop 2000 spectrophotometer (Thermo Scientific, Waltham, MA, USA).

PCR reactions were performed using primers ITS4 and ITS5 [20] in 50 µL of reaction mix (∼50 ng of DNA, 0.25 µM each primer, 0.1 µM of each dNTP, 1 U of Taq DNA Polymerase, 1X PCR buffer, and 0.75 mM MgCl_2_) using a Mastercycler Ep Gradient S (Eppendorf, Hamburg, Germany) set at 94 °C for 3 min; 94 °C for 30 s, 56 °C for 30 s, and 72 °C for 45 s repeated 35 times; and ending with 10 min of extension at 72 °C. PCR products were purified with Amicon Ultra-0.5 mL 100 k 96 PK centrifugal filters (Merck Millipore, Burlington, MA, USA) and prepared for Sanger sequencing using a BigDye Terminator v3.1 Cycle Sequencing Kit (Applied Biosystems, Waltham, MA, USA) according to the manufacturer’s instructions. Libraries were then sequenced on a 3500 Genetic Analyzer (Applied Biosystems, Waltham, MA, USA) in both directions.

Sequences were merged and manually curated using ChromasPro version 1.7.6, and grouped in bins containing identical sequences defined as sequence types (STs) [21]. Each ST was preliminarily identified by querying the GenBank database using the BLASTn tool. Then, each ST was further compared with reference sequences of the same genus to enable their identification with the highest possible level of accuracy. Reference sequences were downloaded from the NCBI Reference Sequence Database and/or selected according to specific taxonomic studies [22,23,24,25,26,27,28]. STs and reference sequences for each fungal genus were aligned using MUSCLE [29], trimmed to the same length, and used to build a phylogenetic tree through the Maximum Likelihood method (Tamura-Nei model, 1000 bootstraps) in MEGA7 [30].

### 2.3. Metabarcoding Characterization of Fungal Communities

We characterized the composition of the whole fungal community in our flour samples using metabarcoding. DNA was extracted from ∼25 mg of flour using the DNeasy Plant Mini Kit (Qiagen, Venlo, Netherlands) according to the manufacturer’s instructions, quantified using a Nanodrop 2000 spectrophotometer (Thermo Scientific, Waltham, MA, USA), and normalized to 50 ng/µL using ultrapure water. The ITS2 region from the fungal rRNA was amplified using the primers ITS3_KYO2 and ITS4 [31] modified to include Illumina overhang adaptors. PCRs were performed in 25 µL of reaction mix (∼50 ng of DNA, 0.5 µM each primer, 1X KAPA Biosystems HiFi HotStart ReadyMix, and nuclease-free water) using a Mastercycler Ep Gradient S (Eppendorf, Hamburg, Germany) set at 95 °C for 3 min; 98 °C for 30 s, 55 °C for 30 s, and 72 °C for 30 s repeated 35 times; and ending with 10 min of extension at 72 °C. Amplifications were performed in technical triplicate, in order to reduce the stochastic variability during amplification. A non-template control in which nuclease-free water (replacing target DNA) was included in all PCR assays, and all the reactions in that bulk were discarded if the no-template control showed amplification. PCR products were then purified with Agencourt Ampure XP SPRI beads (Beckman Coulter Inc., Brea, CA, USA), and 1 µL of the purified amplicons was used for a second PCR to integrate Illumina adaptors using the Nextera XT index Kit (Illumina, San Diego, CA, USA). Amplicons were purified a second time as reported above, quantified using a Qubit 3.0 Fluorometer (Thermo Scientific, Waltham, MA, USA), and pooled at equimolar ratio. The pooled library was then sequenced on a Illumina MiSeq instrument using the 300PE chemistry (Illumina, San Diego, CA, USA).

Demultiplexed forward and reverse reads were merged using the PEAR 0.9.1 [32]. Data handling was carried out using QIIME 1.9 [33], quality-filtering reads, binning operational taxonomic units (OTUs) with a 97% cut-off, and discarding chimeric sequences using VSEARCH [34] with default parameters. All non-fungal OTUs were discarded using ITSx [35], and taxonomy was assigned using the BLAST method by querying the UNITE database (v. 8.0) [36]. Singletons and OTUs coming from amplification of chloroplast DNA were discarded as well.

## 3. Results

### 3.1. Isolation and Identification of Fungi

Fungal cultures were obtained from all the samples. Wholemeal flours yielded the highest number of CFU (527–1840 CFU/g), followed by type “00” (80–173 CFU/g) and type “0” (53–80 CFU/g). The genus *Penicillium* was the most diverse of all investigated samples (Table 1) with 17 different STs and and average of 104 CFU/g of flour. This genus was particularly abundant in wholemeal samples (average 383 CFU/g). Phylotypes were either associate to a single species (*Penicillium aurantiogriseum*, *P. verrucosum*, *P. griseofulvum*, *P. brevicompactum*, and *P. citrinum*) or to two or more reference species with identical or very similar ITS sequences (Table 1). Furthermore, four STs (PEN 1, PEN5, PEN7, and PEN16) were only identified at the level of genus.

The genus *Aspergillus* was isolated from all samples of flour type “00” with a concentration ranging from 7 and 20 CFU/g. The analysis of sequences enabled the identification of two STs associated with five different *Aspergillus* species (Table 1). The genus *Alternaria* was represented by three STs and was isolated from all types of flours but not in all samples. The genus *Cladosporium* was found in 2 out of 3 samples of type “0” and in all of “00”. The genus was represented by four STs clustering in two phylotypes both associated with several different reference species due to the low genetic variability within ITS regions of related *Cladosporium* species (Table 1).

Other fungi were detected with a low abundance and did not show a clear specific association to any flour types. These fungi were identified as *Arthrinium arundinis*, *Epicoccum nigrum*, *Fusarium oxysporum*, and *Mucor circinelloides* (Table 1). Overall, 34 different fungal STs were identified, and the higher fungal diversity in terms of both fungal genera and STs was detected in type “00” as compared to type “0” and wholemeal (Table 1).

### 3.2. Metabarcoding Characterization of Fungal Communities

**Figure 1:**
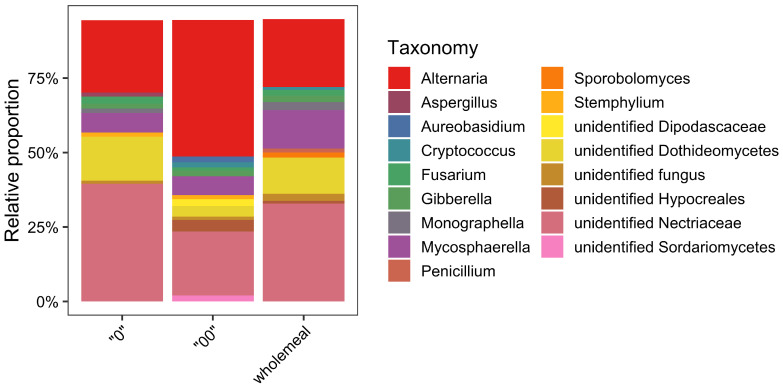
Fungal community of wheat flour samples (type “0”, “00” and wholemeal) according to results of metabarcoding analyses. Fungal genera with a relative abundance ≤1% are not reported.

Following quality trimming, denoising, and chimera removal, 244,189 high quality sequencing reads were obtained from nine samples and assigned to 602 (type “00”), 417 (type “0”), and 301 OTUs (wholemeal). Members of the phylum Ascomycota dominated in all samples and accounted for 94.8% of the total number of detected sequences followed by Basidiomycota (3.3%) and unidentified fungi (1.4%). Within the phylum Ascomycota, Dothideomycetes (52.5%), and Sordariomycetes (39.4%) were found to be the most representative classes. At the level of genus, more than 130 different taxa were identified across all investigated samples. Among these, 17 had a relative abundance ≥1%. The genus *Alternaria* was the most abundant (31%) in all investigated samples (Figure 1). This genus was followed by unidentified Nectriaceae (28.4%), unidentified Dothideomycetes (8.8%), *Mycosphaerella* (8.4%), and *Fusarium* (3.6%). Overall, all genera detected using the culturing method were also detected in metabarcoding analyses although data about the relative abundance were not consistent between the two methods (Figure 1). In particular, the genus *Penicillium*, which was largely the most abundant using the traditional isolation method had an overall relative abundance of 0.8% in metabarcoding analyses (Figure 1), with the highest values in whole meal samples (1.8%).

## 4. Discussion

In this study, we used two different approaches (culture-dependent and -independent) to characterize the fungal microbiome associated with different types of wheat flours. Our results suggest the presence of a wide diversity of fungal species that can be isolated from these products, with several taxa being potential producers of mycotoxins. In addition, we were able to isolate a higher number of CFU from wholemeal flour (527–1840 CFU/g) compared to “00” (80–240 CFU/g) and “0” (53–80 CFU/g) types. This might be the result of the product transformation, as wholemeal flour contains grain elements that are removed in the other two flour types, and this might reduce the whole microbial load.

While in our study we focused on studying the whole fungal community of different types of wheat flour, previous studies mostly focused on identifying mycotoxigenic fungal species [37,38]. For example, Weidenbörner et al. [39] isolated 51 fungal species belonging to 14 different genera from whole and white wheat flour, where species *Aspergillus* were the dominant members of the community, followed by *Penicillium* spp. Similar results were observed in wheat flour [40], cereals used as feed [41], maize flours [42], and pearl millet [43]. On freshly harvested wheat grains, *Alternaria*, *Fusarium* and *Epicoccum* resulted the most common members of the fungal community [44], while Covarelli et al. [45] found that *Fusarium* was the most abundant fungal genus in grains of durum wheat. These examples show that the fungal microbiome associated with grains and flours is quite diverse and variable, even when we look only at the portion that can be isolated and cultivated in vitro.

Our results suggest that *Penicillium* was the most abundant genus within the flour-associated mycobiome, followed by *Cladosporium*, *Aspergillus* and *Alternaria*. Similar results were obtained on whole wheat and corn flour [46], although they contrast with other previous studies [39,40,41,42,43,44,45], while this might seem contradictory, these contrasting results might be the results of contaminations happening at different steps throughout the production chain. Indeed, *Alternaria*, *Cladosporium*, *Fusarium*, and *Helmintosporium* are more common to contaminate grains in the field, while *Aspergillus*, *Penicillium*, *Mucor*, and *Eurotium*, are more common contaminants during the post-harvest phase [2]. Thus, the predominance of genus *Penicillium* in our samples may indicate that the flour products we used were contaminated by potential mycotoxigenic fungi during the postharvest storage.

Interestingly, most of the fungal genera isolated in our study contain species that can produce mycotoxins [38]. *Penicillium*, for example, was the most abundant in our samples with also the highest diversity of STs, and species of this genus have been previously reported to produce the mycotoxins patulin and ochratoxin A [47]. Indeed, among the various species, we isolated *Penicillium verrucosum*, which is one of the most important ochratoxigenic species [48], *Penicillium griseofulvum* known to produce patulin [47]. We also isolated *Penicillium citrinum*, *Penicillium commune*, and *Penicillium chrysogenum*, potentially producers of citrinin, cyclopiazonic acid and roquefortine C, respectively, [47]. Similarly, members of the genus *Aspergillus* are known to produce mycotoxins such as aflatoxins, ochratoxin A and fumonisins [49]. In our study, we identified a single ST belonging to the genus *Aspergillus*, but the resolution of the marker we used did not allow to distinguish between *Aspergillus oryzae*, *Aspergillus kamarensis* and *Aspergillus fasciculatus*. Although these species are not known to produce mycotoxins [37], the identity of these isolates is still unknown, and perhaps they might be related to *Aspergillus flavus* that is an important aflatoxin producer [50]. Additionally, species of *Alternaria* (*Alternaria alternata* and *Alternaria arborescens*) found in our study in low abundance can produce mycotoxins like alternariol or tenuazonic acid [51,52], but are also fungi that are known pathogens and endophytes of wheat in field [53,54]. Thus, their role in our context is still unclear. In addition, we found other fungi belonging to the genera *Fusarium*, *Cladosporium*, and *Mycosphaerella*, which are known pathogens/endophytes of wheat plants, and none of them has been previously reported to produce mycotoxins. Thus, the fact we were able to isolate them can be just the result of plant-microbe interaction occurring in the field, with no influence on the quality/safety of food products.

These results obtained using classic methods for fungal isolation and cultivation in vitro largely matched those obtained using metabarcoding. Qualitatively, all the fungal genera identified through in vitro cultivation were also found in the metabarcoding dataset. However, the region amplified during metabarcoding library preparation is much shorter compared to the one we used to identify fungal isolates. Thus, we were not able to accurately identify all the OTUs to species level, and we preferred to group them at the genus level to avoid providing incorrect information on their identity. In addition, the metabarcoding dataset uncovered a wider diversity of fungal taxa associated with wheat flours, and this can be the results of two main factors. First, the in vitro isolation has the limit that not all organisms can be cultivated, either because they are difficult to cultivate using standard media or they are not cultivable at all [55,56], while metabarcoding is insensitive to this factor. Second, metabarcoding is insensitive to the viability of the fungal cells, so taxa show up in the dataset regardless if fungal cells are viable or not, while in vitro culturing requires alive cells. In addition to surveying the fungal diversity in a sample, metabarcoding allows to estimate the relative abundance of each microbial taxon within a sample, although this information needs to be handled carefully as the rRNA markers of different fungal taxa are not PCR-amplified with the same efficiency because of different factors (e.g., primer set, PCR reagents, reaction temperature) and this might generate misleading results [57]. Thus, even though metabarcoding is an extremely powerful technique that enables the study of microbiomes, results have to be taken acknowledging these limitations.

This study provides a comprehensive picture of the fungal diversity of the three most used typologies of wheat flour. Using both culture-dependent and -independent techniques, we found the presence of several fungal taxa that can cause harm to consumers. Future research can further expand our results, by testing a wider range of producers, sampling timeframes, production areas, and comparing the mycobiome of wheat grains and flour to understand the source of potential mycotoxigenic fungi. A better understanding of the mycobiome of wheat flours increases our knowledge on the frequency and distribution of mycotoxin producers, with a positive impact on food safety. Consumers are increasingly demanding food with higher quality and safety standards, so it is essential to integrate these state-of-the-art tools into the quality control procedures so that unsuitable food products are quickly identified and removed from the production chain.

## Figures and Tables

**Table 1:** List of sequence types (STs) detected in our flour samples (type “0”, “00” and wholemeal) using a conventional culturing method. Each ST is associated with one or more species according to results of phylogenetic analyses.

Sequence Types (STs)	Associated Species	Flour Type
ALT1, ALT3	*Alternaria* sp.	“0”, “00”, wholemeal
ALT2	*Alternaria infectoria*	“00”
ARTH1	*Arthrinium arundinis*	“0”, “00”
ASP1	*Aspergillus fasciculatus*, *A. kambarensis*, *A. oryzae*	“00”
ASP2	*Aspergillus clavatus*, *A. apicalis*	“00”
CHAET1	*Chaetomium globosum*	“00”
CLA1, CLA2, CLA3, CLA4	*Cladosporium* sp.	“0”, “00”
EPI1	*Epicoccum nigrum*	“00”
FUS1	*Fusarium oxysporum*	“00”
LICH1	*Lichtheimia corymbifera*	“00”
MUC1, MUC3	*Mucor circinelloides*	“00”
MUC2	*Mucor* sp.	“00”
PEN1	*Penicillium* sp.	“0”, wholemeal
PEN8	*Penicillium viridicatum*, *P. polonicum*	“0”
PEN12	*Penicillium aurantiogriseum*	“0”, “00”, wholemeal
PEN7	*Penicillium* sp.	“0”, “00”
PEN9	*Penicillium albocoremium*, *P. thymicola*	“00”
PEN6	*Penicillium verrucosum*	“0”, “00”
PEN15	*Penicillium biforme*, *P. commune*, *P. solitum*	“00”
PEN4	*Penicillium confertum*, *P. flavigenum*	“00”
PEN2, PEN13	*Penicillium allii-sativi*, *P. chrysogenum*	“00”, wholemeal
PEN10, PEN17	*Penicillium griseofulvum*	“0”, “00”, wholemeal
PEN5, PEN16	*Penicillium* sp.	“00”, wholemeal
PEN14	*Penicillium brevicompactum*	“00”
PEN11	*Penicillium citrinum*	“00”
RIZH1	*Rhizopus oryzae*	“00”

## References

[1] Bullerman L.B., Bianchini A. (2009). Food safety issues and the microbiology of cereals and cereal products. Microbiologically Safe Foods.

[2] Doyle M.P., Diez-Gonzalez F., Hill C. (2020). Food Microbiology: Fundamentals and Frontiers.

[3] Miller J.D. (1995). Fungi and mycotoxins in grain: Implications for stored product research. J. Stored Prod. Res..

[4] Laca A., Mousia Z., Díaz M., Webb C., Pandiella S.S. (2006). Distribution of microbial contamination within cereal grains. J. Food Eng..

[5] Bhat R., Ramakrishna Y., Beedu S., Munshi K. (1989). Outbreak of trichothecene mycotoxicosis associated with consumption of mould-damaged wheat products in Kashmir Valley, India. Lancet.

[6] Li F.Q., Luo X.Y., Yoshizawa T. (1999). Mycotoxins (trichothecenes, zearalenone and fumonisins) in cereals associated with human red-mold intoxications stored since 1989 and 1991 in China. Nat. Toxins.

[7] Pestka J.J., Smolinski A.T. (2005). Deoxynivalenol: Toxicology and potential effects on humans. J. Toxicol. Environ. Health Part B.

[8] Škrbić B., Živančev J., Đurišić-Mladenović N., Godula M. (2012). Principal mycotoxins in wheat flour from the Serbian market: Levels and assessment of the exposure by wheat-based products. Food Control.

[9] Amirahmadi M., Shoeibi S., Rastegar H., Elmi M., Mousavi Khaneghah A. (2018). Simultaneous analysis of mycotoxins in corn flour using LC/MS-MS combined with a modified QuEChERS procedure. Toxin Rev..

[10] Dos Santos I.D., Pizzutti I.R., Dias J.V., Fontana M.E.Z., Souza D.M., Cardoso C.D. (2021). Mycotoxins in wheat flour: Occurrence and co-occurrence assessment in samples from Southern Brazil. Food Addit. Contam. Part B.

[11] Cheli F., Pinotti L., Rossi L., Dell’Orto V. (2013). Effect of milling procedures on mycotoxin distribution in wheat fractions: A review. LWT-Food Sci. Technol..

[12] Palpacelli V., Beco L., Ciani M. (2007). Vomitoxin and zearalenone content of soft wheat flour milled by different methods. J. Food Prot..

[13] Boudra H., Le Bars P., Le Bars J. (1995). Thermostability of ochratoxin A in wheat under two moisture conditions. Appl. Environ. Microbiol..

[14] Jackson L.S., Hlywka J.J., Senthil K.R., Bullerman L.B., Musser S.M. (1996). Effects of time, temperature, and pH on the stability of fumonisin B1 in an aqueous model system. J. Agric. Food Chem..

[15] Jackson L.S., Hlywka J.J., Senthil K.R., Bullerman L.B. (1996). Effects of thermal processing on the stability of fumonisin B2 in an aqueous system. J. Agric. Food Chem..

[16] Pineda-Valdes G., Bullerman L.B. (2000). Thermal stability of moniliformin at varying temperature, pH, and time in an aqueous environment. J. Food Prot..

[17] Ryu D., Hanna M.A., Eskridge K.M., Bullerman L.B. (2003). Heat stability of zearalenone in an aqueous buffered model system. J. Agric. Food Chem..

[18] Mesterházy Á., Oláh J., Popp J. (2020). Losses in the grain supply chain: Causes and solutions. Sustainability.

[19] Ntuli V., Mekbib S.B., Asita A., Molebatsi N., Makotoko M., Chatanga P. (2013). Microbial and physicochemical characterization of maize and wheat flour from a milling company, Lesotho. Internet J. Food Saf..

[20] White T.J., Bruns T., Lee S.J.W.T., Taylor J. (1990). Amplification and direct sequencing of fungal ribosomal RNA genes for phylogenetics. PCR Protoc. Guide Methods Appl..

[21] Prigigallo M., Mosca S., Cacciola S., Cooke D., Schena L. (2015). Molecular analysis of *Phytophthora* diversity in nursery-grown ornamental and fruit plants. Plant Pathol..

[22] Abe A., Asano K., Sone T. (2010). A molecular phylogeny-based taxonomy of the genus *Rhizopus*. Biosci. Biotechnol. Biochem..

[23] Comby M., Lacoste S., Baillieul F., Profizi C., Dupont J. (2016). Spatial and temporal variation of cultivable communities of co-occurring endophytes and pathogens in wheat. Front. Microbiol..

[24] Irinyi L., Lackner M., De Hoog G.S., Meyer W. (2016). DNA barcoding of fungi causing infections in humans and animals. Fungal Biol..

[25] Jayasiri S., Hyde K., Jones E., Jeewon R., Ariyawansa H., Bhat J., Camporesi E., Kang J. (2017). Taxonomy and multigene phylogenetic evaluation of novel species in *Boeremia* and *Epicoccum* with new records of *Ascochyta* and *Didymella* (Didymellaceae). Mycosphere.

[26] Walther G., Pawłowska J., Alastruey-Izquierdo A., Wrzosek M., Rodriguez-Tudela J., Dolatabadi S., Chakrabarti A., De Hoog G. (2013). DNA barcoding in Mucorales: An inventory of biodiversity. Pers. Mol. Phylogeny Evol. Fungi.

[27] Crous P.W., Groenewald J.Z. (2013). A phylogenetic re-evaluation of Arthrinium. IMA Fungus.

[28] Sandoval-Denis M., Guarnaccia V., Polizzi G., Crous P. (2018). Symptomatic Citrus trees reveal a new pathogenic lineage in Fusarium and two new Neocosmospora species. Pers.-Mol. Phylogeny Evol. Fungi.

[29] Edgar R.C. (2004). MUSCLE: Multiple sequence alignment with high accuracy and high throughput. Nucleic Acids Res..

[30] Kumar S., Stecher G., Tamura K. (2016). MEGA7: Molecular evolutionary genetics analysis version 7.0 for bigger datasets. Mol. Biol. Evol..

[31] Toju H., Tanabe A.S., Yamamoto S., Sato H. (2012). High-coverage ITS primers for the DNA-based identification of ascomycetes and basidiomycetes in environmental samples. PLoS ONE.

[32] Zhang J., Kobert K., Flouri T., Stamatakis A. (2014). PEAR: A fast and accurate Illumina Paired-End reAd mergeR. Bioinformatics.

[33] Caporaso J.G., Lauber C.L., Walters W.A., Berg-Lyons D., Huntley J., Fierer N., Owens S.M., Betley J., Fraser L., Bauer M. (2012). Ultra-high-throughput microbial community analysis on the Illumina HiSeq and MiSeq platforms. ISME J..

[34] Rognes T., Flouri T., Nichols B., Quince C., Mahé F. (2016). VSEARCH: A versatile open source tool for metagenomics. PeerJ.

[35] Bengtsson-Palme J., Ryberg M., Hartmann M., Branco S., Wang Z., Godhe A., De Wit P., Sánchez-García M., Ebersberger I., De Sousa F. (2013). Improved software detection and extraction of ITS1 and ITS 2 from ribosomal ITS sequences of fungi and other eukaryotes for analysis of environmental sequencing data. Methods Ecol. Evol..

[36] Nilsson R.H., Larsson K.H., Taylor A.F.S., Bengtsson-Palme J., Jeppesen T.S., Schigel D., Kennedy P., Picard K., Glöckner F.O., Tedersoo L. (2019). The UNITE database for molecular identification of fungi: Handling dark taxa and parallel taxonomic classifications. Nucleic Acids Res..

[37] Frisvad J.C., Thrane U., Samson R.A. (2007). Mycotoxin producers. Food Mycology.

[38] Moretti A., Logrieco A.F., Susca A. (2017). Mycotoxins: An underhand food problem. Mycotoxigenic Fungi.

[39] Weidenbörner M., Wieczorek C., Appel S., Kunz B. (2000). Whole wheat and white wheat flour—The mycobiota and potential mycotoxins. Food Microbiol..

[40] Alhussaini M.S. (2013). Mycobiota of wheat flour and detection of *α*-amylase and L-asparaginase enzymes. Life Sci. J..

[41] Hassan Z.U., Al-Thani R.F., Migheli Q., Jaoua S. (2018). Detection of toxigenic mycobiota and mycotoxins in cereal feed market. Food Control.

[42] Alborch L., Bragulat M., Castellá G., Abarca M., Cabañes F. (2012). Mycobiota and mycotoxin contamination of maize flours and popcorn kernels for human consumption commercialized in Spain. Food Microbiol..

[43] Hafez S.I.I.A., Sater M.A.A., Hussein N.A.G., Amery E.A.W. (2021). Fungal diversity associated with pearl millet (*Pennisetum glaucum* L.) grains from Taiz governorate, Yemen and their amylase production. J. Microbiol. Biotechnol. Food Sci..

[44] Tralamazza S.M., Bemvenuti R.H., Zorzete P., de Souza Garcia F., Corrêa B. (2016). Fungal diversity and natural occurrence of deoxynivalenol and zearalenone in freshly harvested wheat grains from Brazil. Food Chem..

[45] Covarelli L., Beccari G., Prodi A., Generotti S., Etruschi F., Juan C., Ferrer E., Mañes J. (2015). *Fusarium* species, chemotype characterisation and trichothecene contamination of durum and soft wheat in an area of central Italy. J. Sci. Food Agric..

[46] Dos Santos J.L.P., Bernardi A.O., Morassi L.L.P., Silva B.S., Copetti M.V., Sant’Ana A.S. (2016). Incidence, populations and diversity of fungi from raw materials, final products and air of processing environment of multigrain whole meal bread. Food Res. Int..

[47] Perrone G., Susca A. (2017). Penicillium species and their associated mycotoxins. Mycotoxigenic Fungi.

[48] Cabañes F.J., Bragulat M.R., Castellá G. (2010). Ochratoxin A producing species in the genus *Penicillium*. Toxins.

[49] Perrone G., Gallo A. (2017). Aspergillus species and their associated mycotoxins. Mycotoxigenic Fungi.

[50] Varga J., Frisvad J.C., Samson R. (2011). Two new aflatoxin producing species, and an overview of *Aspergillus* section Flavi. Stud. Mycol..

[51] Escrivá L., Oueslati S., Font G., Manyes L. (2017). *Alternaria* mycotoxins in food and feed: An overview. J. Food Qual..

[52] Nguyen T.T., Kim J., Jeon S.J., Lee C.W., Magan N., Lee H.B. (2018). Mycotoxin production of *Alternaria* strains isolated from Korean barley grains determined by LC-MS/MS. Int. J. Food Microbiol..

[53] Fernández Pinto V.E., Patriarca A. (2017). Alternaria species and their associated mycotoxins. Mycotoxigenic Fungi.

[54] Ofek-Lalzar M., Gur Y., Ben-Moshe S., Sharon O., Kosman E., Mochli E., Sharon A. (2016). Diversity of fungal endophytes in recent and ancient wheat ancestors *Triticum dicoccoides* and *Aegilops sharonensis*. FEMS Microbiol. Ecol..

[55] Al-Sadi A.M., Al-Mazroui S., Phillips A. (2015). Evaluation of culture-based techniques and 454 pyrosequencing for the analysis of fungal diversity in potting media and organic fertilizers. J. Appl. Microbiol..

[56] van Elsas J.D., Duarte G.F., Keijzer-Wolters A., Smit E. (2000). Analysis of the dynamics of fungal communities in soil via fungal-specific PCR of soil DNA followed by denaturing gradient gel electrophoresis. J. Microbiol. Methods.

[57] Abdelfattah A., Li Destri Nicosia M.G., Cacciola S.O., Droby S., Schena L. (2015). Metabarcoding analysis of fungal diversity in the phyllosphere and carposphere of olive (*Olea europaea*). PLoS ONE.

